# Acute type A aortic dissection features and outcomes in octogenarians: a propensity score analysis

**DOI:** 10.1093/icvts/ivae038

**Published:** 2024-03-20

**Authors:** You Kyeong Park, Jae Hang Lee, Kang Min Kim, Joon Chul Jung, Hyoung Woo Chang, Dong Jung Kim, Jun Sung Kim, Cheong Lim, Kay-Hyun Park

**Affiliations:** Department of Thoracic and Cardiovascular Surgery, Seoul National University Bundang Hospital, Seoul National University College of Medicine, Seoul, Republic of Korea; Department of Thoracic and Cardiovascular Surgery, Seoul National University Bundang Hospital, Seoul National University College of Medicine, Seoul, Republic of Korea; Department of Thoracic and Cardiovascular Surgery, Seoul National University Bundang Hospital, Seoul National University College of Medicine, Seoul, Republic of Korea; Department of Thoracic and Cardiovascular Surgery, Seoul National University Bundang Hospital, Seoul National University College of Medicine, Seoul, Republic of Korea; Department of Thoracic and Cardiovascular Surgery, Seoul National University Bundang Hospital, Seoul National University College of Medicine, Seoul, Republic of Korea; Department of Thoracic and Cardiovascular Surgery, Seoul National University Bundang Hospital, Seoul National University College of Medicine, Seoul, Republic of Korea; Department of Thoracic and Cardiovascular Surgery, Seoul National University Bundang Hospital, Seoul National University College of Medicine, Seoul, Republic of Korea; Department of Thoracic and Cardiovascular Surgery, Seoul National University Bundang Hospital, Seoul National University College of Medicine, Seoul, Republic of Korea; Department of Thoracic and Cardiovascular Surgery, Seoul National University Bundang Hospital, Seoul National University College of Medicine, Seoul, Republic of Korea

**Keywords:** Aortic dissection, Octogenarian, Propensity score

## Abstract

**OBJECTIVES:**

The clinical characteristics and early outcomes of surgical repair in octogenarians with acute type A aortic dissection were compared with those in nonoctogenarians.

**METHODS:**

All patients who underwent emergency surgical repair for acute type A aortic dissection in our institution between 2003 and 2022 were included in this study. The patients were divided into an octogenarian group and a nonoctogenarian group. The patients in the 2 groups were propensity score matched at a ratio of 1:1. Before matching, the baseline characteristics were compared between 2 groups. The major complication and 30-day mortality rates were compared in the matched population.

**RESULTS:**

A total of 495 patients were screened, and 471 were included in the analysis, with 48 in the octogenarian group and 423 in the nonoctogenarian group. Before matching, DeBakey type II dissection was significantly more prevalent in the octogenarians (42% vs 14% in the octogenarians and nonoctogenarians, respectively, *P *<* *0.001). Additionally, intramural haematomas (39.6% vs 14.4%, *P *<* *0.001) were more prevalent in the octogenarians. However, severe aortic regurgitation (4.2% vs 15.4%, *P *=* *0.046) and root enlargement (0% vs 13.7%, *P *=* *0.009) were less prevalent in the octogenarians. After matching (36 pairs), the incidence of postoperative delirium was higher in the octogenarians (56% vs 25%, *P *=* *0.027). However, there were no significant differences in 30-day and in-hospital mortality rates, intensive care unit stay or major complications, including stroke, paraplegia, respiratory complications, mediastinitis and haemodialysis.

**CONCLUSIONS:**

The octogenarians with acute type A aortic dissection had higher incidences of DeBakey type II dissection and intramural haematomas and lower incidences of severe aortic regurgitation and aortic root enlargement than the nonoctogenarians. Being an octogenarian was not associated with an increased risk of early major complications or mortality after surgery for acute type A aortic dissection.

**Figure:**
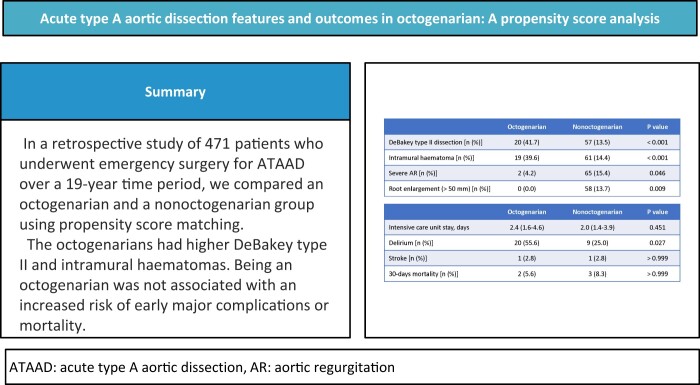


## INTRODUCTION

**Figure 1:**
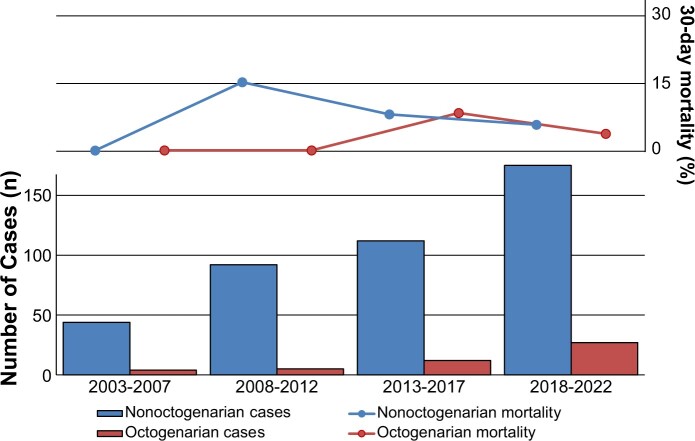
Trends in surgical repair of acute type A aortic dissection and associated 30-day mortality rates.

Acute type A aortic dissection (ATAAD) is a severe life-threatening aortic condition that requires emergency surgical repair [1]. The mortality rate of patients who undergo emergency surgical repair for ATAAD has significantly decreased as surgical techniques, anaesthesia and preoperative medical treatment have improved [2]. However, as the proportion of ageing adults increases worldwide, the number of elderly patients with ATAAD has steadily increased (Fig. 1). However, there are still major concerns regarding surgical intervention for ATAAD in elderly patients, especially octogenarians. According to the International Registry of Acute Aortic Dissection, surgical intervention was not performed in 28% of patients with ATAAD because of advanced age, presence of comorbidities, refusal, intramural haematoma or death before planned surgery [1]. Sometimes, obtaining agreement from octogenarian patients with ATAAD and their families is challenging. Although recent studies have shown that outcomes have improved in octogenarians with ATAAD, the in-hospital mortality rate in this group ranges from 25% to 37%, which is still worse than that in younger patients [3, 4]. Previous studies have shown that elderly patients have higher surgical risks [4–7]. However, it is not clear whether age is a significant risk factor for poor early outcomes even after controlling for baseline comorbidities.

Consequently, the aims of this study were to investigate the clinical characteristics of octogenarians requiring surgical repair for ATAAD and compare the early outcomes of surgical repair between octogenarians and nonoctogenarians. In addition, we also investigated whether age acts as an independent risk factor for mortality and complications by controlling for baseline comorbidities and preoperative status.

## PATIENTS AND METHODS

### Ethical statement

The study was approved by the institutional review board, Seoul National University Bundang Hospital (IRB No. B-2211-792-101) on 24 October 2022. Individual patient consent was waived considering the retrospective nature of this study.

### Patients

**Figure 2:**
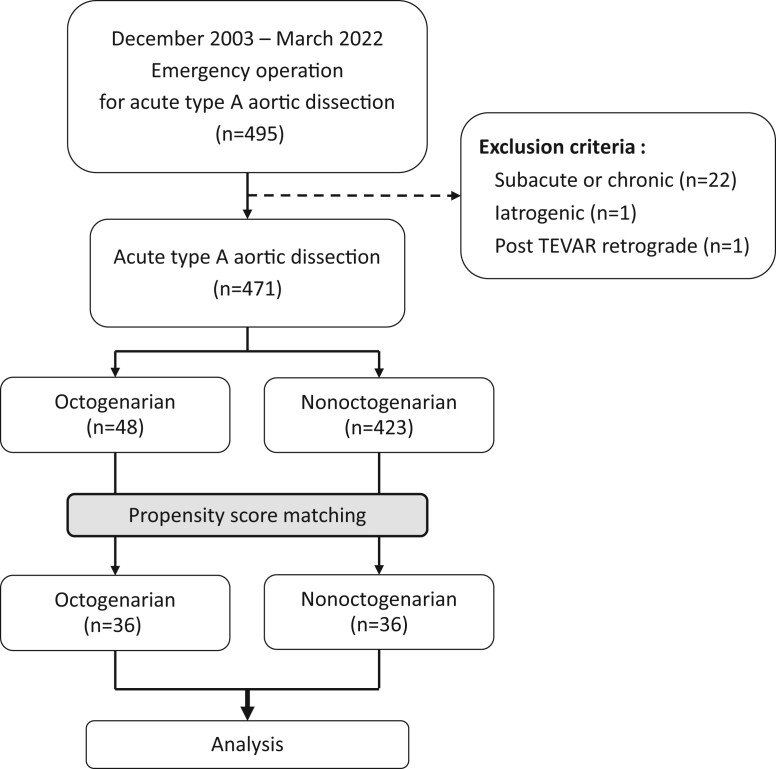
Flow diagram of study.

Adult patients who underwent emergency surgical repair for ATAAD from 2003 to 2022 in our institution were initially screened. The exclusion criteria are listed in Fig. 2. Patients with operative findings suggesting subacute or chronic aortic dissection were excluded. Patients were divided into 2 groups based on their ages: the octogenarian group, patients who were 80 years or older and the nonoctogenarian group, patients who were younger than 80 years. Electronic medical records were reviewed for patient demographics and preoperative status. Operative details and clinical outcomes were compared between the 2 groups.

### Surgical indication and operative techniques

Surgical repair is generally indicated in all patients with ATAAD to prevent complications such as aortic rupture or malperfusion of vital organs. In cases of patients with malperfusion, emergency central aortic repair was performed as a first-line approach. If malperfusion persisted even after central repair, endovascular treatment or bypass surgery was performed on the respective malperfused vessels. For patients with intramural haematoma, emergent surgery was performed if the intramural haematoma thickness was >10 mm, the ascending aortic diameter was >45 mm or if pericardial effusion was present. Most of the operations were performed through standard median sternotomy. Arterial cannulation sites were determined considering the aortic condition. The ascending aorta was preferred as the primary cannulation site when feasible. The right axillary artery was used as a perfusion route in patients who were unsuitable for ascending aorta cannulation due to total collapse of the true lumen or a high embolic risk. The femoral artery was rarely cannulated except in cases of unstable vital signs. After venous cannulation and initiation of cardiopulmonary bypass (CPB), systemic cooling was applied.

Deep hypothermic circulatory arrest was performed at a nasopharyngeal temperature of 20–22°C, and then, the aorta was opened from the sinotubular junction to the level of the innominate artery ostium, and the aortic arch and brachiocephalic branches were explored. Bilateral selective cerebral perfusion was used in partial or total arch replacement, while hemiarch replacement was usually performed with or without unilateral selective cerebral perfusion. The extent of distal repair was decided based on the location of the entry tear. If the entry tear was in the ascending aorta or lesser curvature of the aortic arch, hemiarch or partial aortic arch replacement was performed. Total arch replacement was considered for patients with an aortic arch or proximal descending thoracic aorta that was >40 mm, patients with extensive dissection involving the aortic arch branches, patients with genetic or familial diseases and patients who were relatively younger. In total arch replacement, open distal anastomosis using the surgical elephant trunk technique was preferred. In recent years, the frozen elephant trunk technique has been used in patients with complications due to true lumen compression of the descending aorta or a primary entry tear in the proximal descending thoracic aorta.

Aortic root procedures, including the Bentall operation and valve-sparing aortic root replacement, were considered for patients with sinus of Valsalva enlargement, patients with an intimal tear in the aortic root and patients with a genetic or familial disease. Selection between the 2 techniques was based on the aortic valve leaflets, the age of the patient and the surgeon’s preference.

### Clinical outcomes

The primary outcome was 30-day mortality. Secondary outcomes included in-hospital mortality, intensive care unit stay, operation time, CPB time, aorta cross-clamp time, postoperative complications including stroke, paraplegia, delirium, respiratory complications [prolonged ventilation (>48 h), reintubation, pneumonia, tracheostomy], vocal cord palsy, reoperation due to bleeding, mediastinitis and haemodialysis. Stroke was defined as any newly developed central neurological deficit with significant lesions on neurological imaging. Paraplegia was defined as impairment in motor or sensory function of the lower extremities that occurs as a result of injury to the spinal cord. Delirium was defined as consciousness disturbances, disorders of concentration or cognition such as disorientation, memory disturbances and hallucinations [8]. Vocal cord palsy was diagnosed by laryngoscopy. Mediastinitis was defined as infection involving the mediastinum that was microbiologically proven.

### Statistical analysis

For statistical analysis, SPSS 26 (SPSS Inc. Chicago, IL, USA) was used. Continuous variables are presented as medians [interquartile ranges (IQRs)], and categorical variables are presented as numbers and percentages in parentheses. One-to-one propensity score matching using the nearest neighbour method was performed to overcome the differences in baseline characteristics between the octogenarians and the nonoctogenarians. The matching variables are listed in Table 1: patient demographics, baseline comorbidities, preoperative status, preoperative aortic conditions (including DeBakey type, intramural haematoma, location of tear) and the extent of aortic repair. Logistic regression analysis was performed to estimate the propensity score, and matching was established using a calliper with a width equal to 0.18 of the standard deviation of the logit of the propensity score. The appropriateness of the matching was evaluated with the standardized mean difference. After propensity score matching, 36 pairs of octogenarians and nonoctogenarians were made. The standardized mean differences of all the matching variables were <15% after matching. Before matching, the differences in the characteristics between the 2 groups were tested using independent sample *t*- or chi-squared tests (or Fisher’s exact test when necessary). After matching, the differences between groups were tested using a paired *t* test or McNemar’s test. Kaplan–Meier survival analysis and the log-rank test were used to determine overall survival rates before matching. Additionally, a further stratified log-rank test was performed after matching. Univariate and multivariate logistic regression analyses were used to estimate the factors influencing early mortality. The predictive factors with a *P*-value of <0.20 in the univariate analysis and being octogenarian were included in the multivariable regression for adjustment. A *P*-value of <0.05 was considered statistically significant.

**Table 1:** Baseline characteristics before and after propensity score matching

	Octogenarian (*n* = 48)	Nonoctogenarian (*n* = 423)	SMD (%)	*P* value	Octogenarian (*n* = 36)	Nonoctogenarian (*n* = 36)	SMD (%)	*P* value
Age	83 (81–85.8)	58 (48–69)			83 (81–84)	69.5 (59–76)		<0.001
Sex, male	7 (14.6)	243 (57.5)	121.5	<0.001	7 (19.4)	7 (19.4)	0.0	>0.999
BMI	22.4 (20.0–24.6)	24.7 (22.3–27.2)	89.1	<0.001	22.7 (20.7–25.3)	22.5 (20.8–26.1)	11.4	0.678
Comorbidity								
Hypertension	40 (83.3)	277 (65.5)	47.9	0.014	31 (86.1)	30 (83.3)	7.4	>0.999
Diabetes mellitus	9 (18.8)	33 (7.8)	28.1	0.027	5 (13.9)	6 (16.7)	7.0	>0.999
Hyperlipidaemia	9 (18.8)	52 (12.3)	16.5	0.253	9 (25.0)	10 (27.8)	7.0	>0.999
Cerebrovascular disease	9 (18.8)	27 (6.4)	31.7	0.006	4 (11.1)	3 (8.3)	7.0	>0.999
ESRD	1 (2.1)	7 (1.7)	3.0	0.580	0 (0.0)	0 (0.0)	0.0	
COPD	1 (2.1)	13 (3.1)	6.9	> 0.999	1 (2.8)	1 (2.8)	0.0	>0.999
Coronary artery disease	7 (14.6)	32 (7.6)	19.9	0.192	6 (16.7)	5 (13.9)	7.8	>0.999
Marfan syndrome	0 (0.0)	41 (9.7)		0.015	0 (0.0)	3 (8.3)		0.250
Preoperative status								
Preoperative CPR	2 (4.2)	31 (7.3)	15.8	0.560	2 (5.6)	2 (5.6)	0.0	>0.999
Cardiac tamponade	12 (25.0)	63 (14.9)	23.3	0.093	8 (22.2)	7 (19.4)	6.3	>0.999
Severe AR	2 (4.2)	65 (15.4)	56.1	0.046	2 (5.6)	3 (8.3)	13.8	>0.999
Root enlargement (>50 mm)	0 (0.0)	58 (13.7)	38.0	0.009	0 (0.0)	2 (5.6)		0.500
DeBakey type II dissection	20 (41.7)	57 (13.5)	57.2	<0.001	11 (30.6)	12 (33.3)	5.6	>0.999
Intramural haematoma	19 (39.6)	61 (14.4)	51.5	<0.001	12 (33.3)	11 (30.6)	5.6	>0.999
Location of entry tear								
Ascending aorta	34 (70.8)	246 (58.2)	27.9	0.120	24 (66.7)	23 (63.9)	6.0	>0.999
Aortic arch	13 (27.1)	132 (31.2)	9.3	0.623	8 (22.2)	10 (27.8)	12.4	0.727
Operative details								
Aortic root procedure	2 (4.2)	112 (26.5)	111.7	0.001	2 (5.6)	2 (5.6)	0.0	>0.999
Total arch replacement	18 (37.5)	150 (35.5)	4.2	0.874	16 (44.4)	15 (41.7)	5.7	>0.999

Values are presented as numbers (%) or medians (IQRs).

AR: aortic regurgitation; BMI: body mass index; COPD: chronic obstructive pulmonary disease; CPR: cardiopulmonary resuscitation; ESRD: end-stage renal disease; IQRs: interquartile ranges; SMD: standardized mean difference.

## RESULTS

### Baseline characteristics

A total of 495 patients were screened, and 471 patients were enrolled for analysis. 48 patients were included in the octogenarian group [median age 83 (IQR, 81–85.8) years; range 80–89 years]; and 423 patients were included in the nonoctogenarian group [median age 58 (IQR, 48–69) years; range 20–79 years]. The baseline characteristics and preoperative status of patients in each group are shown in Table 1. Before matching, there were fewer male octogenarians (14.6% vs 57.5% in the octogenarian and nonoctogenarian groups, respectively, *P *<* *0.001). In the octogenarian group, there were significantly more patients with a history of hypertension (83.3% vs 65.5%, *P *=* *0.014), diabetes mellitus (18.8% vs 7.8%, *P *=* *0.027) and cerebrovascular accident (18.8% vs 6.4%, *P *=* *0.006). Forty-one patients with Marfan syndrome were included in the nonoctogenarian group, and there were no Marfan syndrome patients in the octogenarian group (*P *=* *0.015).

### Clinical features

The incidence of preoperative cardiac tamponade was slightly higher in the octogenarian group (25.0% vs 14.9% in the octogenarian and nonoctogenarian groups, respectively, *P *=* *0.093). DeBakey type II dissection (41.7% vs 13.5% in octogenarian and nonoctogenarian groups, respectively, *P *<* *0.001) and intramural haematoma (39.6% vs 14.4% in octogenarian and nonoctogenarian groups, respectively, *P *<* *0.001) were more frequently found in the octogenarians. However, severe aortic regurgitation and root enlargement were less prevalent in the octogenarian group. The location of the tear was more frequently in the ascending aorta in the octogenarian group, but it did not reach statistical significance (70.8% in octogenarian and 58.2% in nonoctogenarian groups, *P *=* *0.120). The preoperative neurologic deficits from all causes and malperfusion of vital organs are described in Table 2.

**Table 2:** Clinical presentation before and after propensity score matching

	Before matching			After matching		
	Octogenarian (*n* = 48)	Nonoctogenarian (*n* = 423)	*P* value	Octogenarian (*n* = 36)	Nonoctogenarian (*n* = 36)	*P* value
Neurologic deficit	8 (16.7)	87 (20.6)	0.523	7 (19.4)	6 (16.7)	>0.999
Malperfusion						
Coronary	0 (0.0)	36 (8.5)	0.039	0 (0.0)	0 (0.0)	>0.999
Brain	5 (10.4)	72 (17.0)	0.241	5 (13.9)	6 (16.7)	>0.999
Mesenteric	2 (4.2)	41 (9.7)	0.292	2 (5.6)	3 (8.3)	>0.999
Lower limb	2 (4.2)	57 (13.5)	0.065	2 (5.6)	5 (13.9)	0.375
Renal	2 (4.2)	55 (13.0)	0.075	2 (5.6)	6 (16.7)	0.219

Values are presented as numbers (%).

### Operative details

The operative details are presented in Tables 1 and 3. In the unmatched comparison, there was no statistically significant difference in the distal extent of the repair. Aortic root procedures, including the Bentall operation and valve-sparing aortic root replacement, were more frequently performed in the nonoctogenarians (4.2% in the octogenarian group and 26.5% in the nonoctogenarian group, *P *=* *0.001). After propensity score matching, there were no statistically significant differences in either the distal extent of the repair or the aortic root procedure (Table 1). Before propensity score matching, the operative, CPB and aorta cross-clamp times were significantly shorter in the octogenarian group. However, they did not show statistically significant differences after matching.

**Table 3:** Operative details and postoperative outcomes before and after propensity score matching

	Before matching			After matching		
	Octogenarian (*n* = 48)	Nonoctogenarian (*n* = 423)	*P* value	Octogenarian (*n* = 36)	Nonoctogenarian (*n* = 36)	*P* value
Operative details						
Operation time (min)	258 (236-289)	300 (255-350)	<0.001	260 (240-290)	290 (247-334)	0.155
CPB time (min)	139 (125-155)	159 (133-192)	<0.001	143 (126-163)	151 (129-182)	0.423
ACC time (min)	96 (82-114)	107 (84-141)	0.011	99 (85-118)	95 (79-113)	0.689
Complications						
Stroke	1 (2.1)	21 (5.0)	0.715	1 (2.8)	1 (2.8)	>0.999
Permanent neurologic deficit	0 (0.0)	14 (3.3)	0.380	0 (0.0)	1 (2.8)	>0.999
Temporary neurologic deficit	1 (2.1)	8 (1.9)	>0.999	1 (2.8)	0 (0.0)	>0.999
Paraplegia	0 (0.0)	7 (1.7)	>0.999	0 (0.0)	1 (2.8)	>0.999
Delirium	22 (45.8)	109 (25.8)	0.006	20 (55.6)	9 (25.0)	0.027
Respiratory complications						
Prolonged ventilation (>48 h)	14 (29.2)	126 (29.8)	>0.999	10 (27.8)	11 (30.6)	>0.999
Reintubation	6 (12.5)	46 (10.9)	0.807	4 (11.1)	4 (11.1)	>0.999
Pneumonia	4 (8.3)	40 (9.5)	>0.999	2 (5.6)	3 (8.3)	>0.999
Tracheostomy	3 (6.3)	26 (6.1)	>0.999	2 (5.6)	2 (5.6)	>0.999
Vocal cord palsy	3 (6.3)	38 (9.0)	0.786	2 (5.6)	4 (11.1)	>0.687
Reoperation due to bleeding	4 (8.3)	119 (28.1)	0.001	3 (8.3)	9 (25.0)	0.146
Mediastinitis	1 (2.1)	8 (1.9)	> 0.999	1 (2.8)	0 (0.0)	>0.999
Haemodialysis	2 (4.2)	44 (10.4)	0.207	2 (5.6)	0 (0.0)	0.500
Intensive care unit stay (days)	2.5 (1.6-4.6)	2.4 (1.4-4.6)	0.620	2.4 (1.6-4.6)	2.0 (1.4-3.9)	0.451
30-Day mortality	2 (4.2)	36 (8.5)	0.407	2 (5.6)	3 (8.3)	>0.999
In-hospital mortality	2 (4.2)	43 (10.2)	0.296	2 (5.6)	3 (8.3)	>0.999

Values are presented as numbers (%) or medians (IQRs). The differences in characteristics between octogenarians and nonoctogenarians were tested for significance using a paired *t* test for continuous variables and McNemar’s test for categorical variables after matching.

ACC: aortic cross-clamp; CPB: cardiopulmonary bypass; IQRs: interquartile ranges.

### Postoperative outcomes

**Figure 3:**
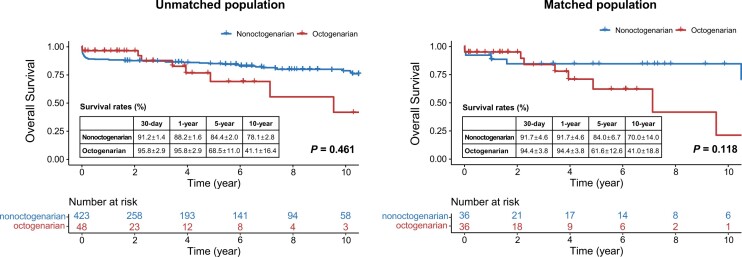
Kaplan–Meier analysis of overall survival rates before and after matching.

The clinical outcomes are presented in Table 3. There was no significant difference in the 30-day mortality rate before or after matching. The overall survival rates are shown in Fig. 3. The length of intensive care unit stay was not significantly different before and after matching. Before and after matching, there were no significant differences between the 2 groups in terms of the incidence of major complications, including stroke, paraplegia, respiratory complications, vocal cord palsy, mediastinitis and haemodialysis. Reoperation due to bleeding was more frequently performed in the nonoctogenarian group before matching. After matching, reoperation due to rebleeding was more frequently performed in the nonoctogenarian group, but it did not reach statistical significance. On the other hand, the incidence of delirium was significantly higher in the octogenarian group before matching, and the difference was statistically significant even after matching.

### Risk factor analysis

Multivariate regression analysis indicated that preoperative cardiopulmonary resuscitation and preoperative cardiac tamponade were significant predictors of early mortality after adjustment for potential confounding factors. DeBakey type II dissection was independently associated with a decreased risk of mortality (odds ratio = 0.165, 95% confidence interval 0.033–0.824, *P *=* *0.028). Being an octogenarian was not an independent predictor of early mortality (odds ratio = 0.571, 95% confidence interval 0.097–3.358, *P *=* *0.535) (Table 4).

**Table 4:** Univariate and multivariate logistic regression analyses of the risk factors for 30-day mortality

	Univariate model		Multivariate model	
	OR (95% CI)	*P* value	OR (95% CI)	*P* value
Octogenarian	0.467 (0.109-2.005)	0.407	0.571 (0.097-3.358)	0.535
Sex, male	1.391 (0.707-2.739)	0.337		
BMI	1.039 (0.955-1.131)	0.378		
Comorbidity				
Hypertension	0.572 (0.292-1.118)	0.099	0.657 (0.305-1.418)	0.285
Diabetes mellitus	0.258 (0.035-1.933)	0.234		
Cerebrovascular disease	1.474 (0.492-4.415)	0.518		
ESRD	1.645 (0.197-13.731)	0.492		
Coronary artery disease	2.273 (0.887-5.826)	0.114	2.096 (0.677-6.487)	0.199
Marfan syndrome	0.266 (0.035-1.987)	0.234		
Preoperative status				
Preoperative CPR	17.797 (7.947-39.853)	<0.001	10.835 (4.465-26.289)	<0.001
Cardiac tamponade	4.054 (2.003-8.208)	<0.001	3.601 (1.502-8.633)	0.004
Severe AR	1.143 (0.459-2.849)	0.773		
Root enlargement (>50 mm)	0.826 (0.282-2.418)	>0.999		
DeBakey type II dissection	0.265 (0.062-1.125)	0.054	0.165 (0.033-0.824)	0.028
Intramural haematoma	0.253 (0.060-1.072)	0.045	0.248 (0.051-1.216)	0.086
Location of entry tear				
Ascending aorta	1.050 (0.533-2.069)	0.888		
Aortic arch	1.041 (0.510-2.126)	0.912		
Operative details				
Aortic root procedure	0.969 (0.445-2.114)	0.938		
Total arch replacement	0.933 (0.464-1.875)	0.845		

AR: aortic regurgitation; BMI: body mass index; CI: confidence interval; CPR: cardiopulmonary resuscitation; ESRD: end-stage renal disease; OR: odds ratio.

In the octogenarian group, 1 patient (81 years old, female) who had neurologic symptoms in the initial presentation died of respiratory complications during postoperative care, and another patient (80 years old, female) who received cardiopulmonary resuscitation during chest opening died of postoperative brain death following cardiac arrest occurred due to tamponade upon entering the operating room. In the nonoctogenarian group after matching, 1 patient (71 years old, female) was presumed to have died of rupture of the false lumen of the descending thoracic aorta, as evidenced by the sudden drainage of arterial blood through the drainage catheter in the left thoracic cavity on the 16th day after surgery. The second patient (49 years old, male) died due to a rupture at the proximal anastomosis site 5 h after the surgery. The last patient (62 years old, female) suffered from low cardiac output syndrome and did not recover despite receiving extracorporeal membrane oxygenation support.

## DISCUSSION

There are 2 main findings in our study. First, the octogenarian patients with ATAAD in our study more frequently had DeBakey type II dissection or an intramural haematoma and more frequently had cardiac tamponade. Second, the outcomes of surgical treatment in octogenarian ATAAD patients did not differ significantly from those of nonoctogenarians in terms of early mortality and major complication rates.

According to a comparative study of patients who were <70 years of age and those ≥70 years by Mehta *et al.* [6], elderly patients showed more severe systemic atherosclerosis and had prior aortic aneurysms more frequently. In the clinical presentation, cardiac murmur was less frequently heard, but the incidences of intramural haematomas and false lumen thrombosis were higher in elderly patients.

In our study population, octogenarians with ATAAD were more frequently categorized as having DeBakey type II dissection and intramural haematomas, and more patients showed cardiac tamponade at initial presentation. These 2 findings might be related to each other. As the pressure of the false lumen in the ascending aorta is usually higher in patients with DeBakey type II dissection than in those with DeBakey type I dissection, the chance of microrupture in the aortic root area could be higher. The higher incidence of DeBakey type II dissection is attributable to the characteristics of aortic pathology in octogenarians. A concomitant penetrating atherosclerotic ulcer (PAU) and aortic wall calcification can have preventive effects on the distal progression of aortic dissection. This is because the progression of the dissection ceases at the site of the PAU or because the dissection cannot propagate beyond aortic wall calcification. Paradoxically, the PAU itself can also precede the development of an intramural haematoma, thus partly explaining the finding that intramural haematomas more frequently occurred in octogenarians [9].

From a surgical perspective, surgical treatment has shown better results than medical treatment in octogenarians with ATAAD [1, 10, 11]. In fact, according to data obtained from the International Registry of Acute Aortic Dissection, old age (>70 years) was considered a risk factor for early mortality from the 1990s to the early 2000s [5, 6]. However, with advances in surgical and anaesthesia techniques in the 2010s, several studies have reported improved surgical outcomes of ATAAD in octogenarians. Kawahito *et al.* [12] reported that the in-hospital mortality rate was 6.3% for octogenarian patients with ATAAD and that the in-hospital mortality and complication rates for surgical repair showed no statistically significant difference from those of their younger counterparts.

However, in their series, the extent of dissection was relatively limited in octogenarians, so the extent of surgical repair was relatively smaller. The authors of these articles recommended limiting the extent of surgery in octogenarians, considering their limited life span, frailty and low reintervention rate. Kondoh *et al.* [13] reported that the 30-day mortality rate was 6.5% and the in-hospital mortality rate was 13% in 90 octogenarians with ATAAD who underwent limited proximal aortic replacement. Their strategy was to only perform isolated ascending aorta replacement whenever possible, and total arch replacement was performed in only 4 out of 90 patients with an aortic arch >60 mm in diameter or a ruptured aortic arch.

Contrary to previous reports, our institute adhered to the centre policy in determining the extent of surgery regardless of patient age, as we had dedicated aortic surgeons. Therefore, the distal extent of surgery did not differ significantly between octogenarians and nonoctogenarians. Our policy is described in the ‘Surgical indication and operative techniques’ section. Although the tear site was more frequently found in the ascending aorta in octogenarians, the distal extent of surgery was similar in the 2 groups. This finding may be explained by the fact that octogenarians more frequently had prior aortic aneurysms. There was no significant difference in the early mortality or major complication rates between the octogenarians and nonoctogenarians despite the similarity in the distal extent of surgical repair. Thus, it might be an option to minimize the extent of surgery to avoid shortening the lifespan of octogenarians with ATAAD, but excluding the intimal tear site is still a reasonable approach in octogenarians when performed by an experienced team.

Aortic root enlargement is one of the most important risk factors for aortic dissection. It has been reported that root enlargement of >50 mm is more common in patients with genetic disorders or bicuspid aortic valves. The increased wall tension in the presence of weakened tissue causes gradual enlargement of the aortic root before dissection occurs [14, 15]. Therefore, before matching, the incidence of aortic root enlargement beyond 50 mm could be higher in nonoctogenarians than in octogenarians, as nonoctogenarians potentially included patients with genetic disorders or bicuspid aortic valves. However, the difference was no longer observed after propensity score matching.

Another concern in determining whether to perform surgery in octogenarians with ATAAD would be neurologic complications during postoperative recovery. As patients could not be thoroughly evaluated before the emergency operation, predisposing factors such as carotid artery stenosis might be undetected at the time of entry into the operating room. In these cases, neurological recovery may be delayed or may not ultimately reach a satisfactory level. Consequently, the chance of prolonged ventilation, tracheostomy or pneumonia could be increased. However, there is a paucity of data supporting this concern. In our study, neurologic complications, including permanent and temporary neurologic deficits, were less common in octogenarians than in nonoctogenarians, although the incidence was very low. This is in line with previous reports [4, 7, 12, 16]. There were no significant differences between the 2 groups in terms of the incidence of respiratory complications in this study. Therefore, we think that these complications can be prevented or overcome with a multidisciplinary approach in the postoperative period.

In our study, only delirium occurred more frequently in octogenarians. Several studies have reported that age is a well-established predictor of delirium [8, 17]. This may be explained by several hypotheses, such as the increased risk of cerebral embolization, decreased cerebral perfusion due to atherosclerosis and the lack of cholinergic reserves. As patients with delirium experienced more adverse events, resulting in an increased 30-day mortality rate, predicting the incidence of delirium by assessing the proven risk factors and taking multicomponent prophylactic measures could decrease the mortality rate in elderly patients.

### Limitations

There are several limitations to our study. Firstly, this is a retrospective and single institutional study based on data obtained over a 19-year period, and there might be changes in practice and surgical approach during this period. Furthermore, the current octogenarians exhibit notably improved physical conditions compared to those from 2 decades ago. Another limitation is potential selection bias. As most of the patients were referred patients, the authors cannot ascertain who were not referred or who refused surgery prior to referral. This effect may have had a greater impact on the octogenarians. Additionally, our study did not employ the proportional hazards assumption, as our focus was on short-term outcomes, particularly analysing the 30-day mortality risk factors. Lastly, using an age cutoff at 80 years does not eliminate the potential influence of different age groups or continuous age on outcomes after surgical repair of ATAAD.

## CONCLUSION

Octogenarians with ATAAD had higher prevalences of DeBakey type II dissection and intramural haematomas as well as fewer cases of aortic root enlargement than nonoctogenarians. The early surgical outcomes showed acceptable results compared with nonoctogenarians before and after matching of the baseline characteristics and preoperative statuses. Therefore, when facing octogenarians with ATAAD, age alone does not have to be considered an exclusion criterion for surgical repair.

## Funding

No funding was received for this study.

**Conflict of interest:** none declared.

## Data Availability

The data supporting the findings in this article will be shared by the corresponding author on reasonable request.

## ABBREVIATIONS

ATAAD: Acute type A aortic dissection
CPB: Cardiopulmonary bypass
IQR: Interquartile range
PAU: Penetrating atherosclerotic ulcer

## References

[1] Hagan PG , Nienaber CA, Isselbacher EM, Bruckman D, Karavite DJ, Russman PL et al The International Registry of Acute Aortic Dissection (IRAD): new insights into an old disease. JAMA 2000;283:897–903. 10685714 10.1001/jama.283.7.897

[2] Pape LA , Awais M, Woznicki EM, Suzuki T, Trimarchi S, Evangelista A et al Presentation, diagnosis, and outcomes of acute aortic dissection: 17-year trends from the International Registry of Acute Aortic Dissection. J Am Coll Cardiol 2015;66:350–8. 26205591 10.1016/j.jacc.2015.05.029

[3] Biancari F , Vasques F, Benenati V, Juvonen T. Contemporary results after surgical repair of type A aortic dissection in patients aged 80 years and older: a systematic review and meta-analysis. Eur J Cardiothorac Surg 2011;40:1058–63. 21561787 10.1016/j.ejcts.2011.03.044

[4] Rylski B , Hoffmann I, Beyersdorf F, Suedkamp M, Siepe M, Nitsch B et al; Multicenter Prospective Observational Study. Acute aortic dissection type A: age-related management and outcomes reported in the German Registry for Acute Aortic Dissection Type A (GERAADA) of over 2000 patients. Ann Surg 2014;259:598–604. 23657079 10.1097/SLA.0b013e3182902cca

[5] Trimarchi S , Eagle KA, Nienaber CA, Rampoldi V, Jonker FH, De Vincentiis C et al; International Registry of Acute Aortic Dissection Investigators. Role of age in acute type A aortic dissection outcome: report from the International Registry of Acute Aortic Dissection (IRAD). J Thorac Cardiovasc Surg 2010;140:784–9. 20176372 10.1016/j.jtcvs.2009.11.014

[6] Mehta RH , O'Gara PT, Bossone E, Nienaber CA, Myrmel T, Cooper JV et al; International Registry of Acute Aortic Dissection (IRAD) Investigators. Acute type A aortic dissection in the elderly: clinical characteristics, management, and outcomes in the current era. J Am Coll Cardiol 2002;40:685–92. 12204498 10.1016/s0735-1097(02)02005-3

[7] Hsu ME , Chou AH, Cheng YT, Lee HA, Liu KS, Chen DY et al Outcomes of acute aortic dissection surgery in octogenarians. J Am Heart Assoc 2020;9:e017147. 32912018 10.1161/JAHA.120.017147 PMC7726989

[8] Bakker RC , Osse RJ, Tulen JH, Kappetein AP, Bogers AJ. Preoperative and operative predictors of delirium after cardiac surgery in elderly patients. Eur J Cardiothorac Surg 2012;41:544–9. 22345177 10.1093/ejcts/ezr031

[9] Sundt TM. Intramural hematoma and penetrating atherosclerotic ulcer of the aorta. Ann Thorac Surg 2007;83:S835–41; discussion S46–50. 17257937 10.1016/j.athoracsur.2006.11.019

[10] Yanagisawa S , Yuasa T, Suzuki N, Hirai T, Yasuda N, Miki K et al Comparison of medically versus surgically treated acute type a aortic dissection in patients <80 years old versus patients >/=80 years old. Am J Cardiol 2011;108:453–9. 21600540 10.1016/j.amjcard.2011.03.067

[11] Hata M , Sezai A, Niino T, Yoda M, Unosawa S, Furukawa N et al Should emergency surgical intervention be performed for an octogenarian with type A acute aortic dissection? J Thorac Cardiovasc Surg 2008;135:1042–6. 18455582 10.1016/j.jtcvs.2007.08.078

[12] Kawahito K , Kimura N, Yamaguchi A, Aizawa K, Misawa Y, Adachi H. Early and late surgical outcomes of acute type A aortic dissection in octogenarians. Ann Thorac Surg 2018;105:137–43. 29054307 10.1016/j.athoracsur.2017.06.057

[13] Kondoh H , Satoh H, Daimon T, Tauchi Y, Yamamoto J, Abe K et al Outcomes of limited proximal aortic replacement for type A aortic dissection in octogenarians. J Thorac Cardiovasc Surg 2016;152:439–46. 27167019 10.1016/j.jtcvs.2016.03.093

[14] Akutsu K. Etiology of aortic dissection. Gen Thorac Cardiovasc Surg 2019;67:271–6. 30689200 10.1007/s11748-019-01066-x

[15] Nienaber CA , Clough RE, Sakalihasan N, Suzuki T, Gibbs R, Mussa F et al Aortic dissection. Nat Rev Dis Primers 2016;2:16071. 27560366 10.1038/nrdp.2016.71

[16] Neri E , Toscano T, Massetti M, Capannini G, Carone E, Tucci E et al Operation for acute type A aortic dissection in octogenarians: is it justified? J Thorac Cardiovasc Surg 2001;121:259–67. 11174731 10.1067/mtc.2001.112205

[17] Rudolph JL , Ramlawi B, Kuchel GA, McElhaney JE, Xie D, Sellke FW et al Chemokines are associated with delirium after cardiac surgery. J Gerontol A Biol Sci Med Sci 2008;63:184–9. 18314455 10.1093/gerona/63.2.184 PMC2735245

